# ATP and appetite: mitochondrial efficiency predicts meal size and the time until next feeding in common minnows

**DOI:** 10.1098/rsos.250557

**Published:** 2025-07-09

**Authors:** Ailsa Bell, Neil B. Metcalfe, Neal J. Dawson

**Affiliations:** ^1^ School of Biodiversity, One Health and Veterinary Medicine, College of Medical, Veterinary & Life Sciences, University of Glasgow, Glasgow, UK

**Keywords:** behaviour, fish, ectotherms, metabolism, dynamic action, digestion, feed efficiency

## Abstract

The predicted collapses of trophic food webs and diminished food availability, likely to be exacerbated by the effects of climate change, are of particular concern for aquatic species. Feeding behaviour influences how aquatic organisms respond to these conditions, and while the efficiency of various mitochondrial traits have been linked to growth outcomes and metabolic traits in fish, the role of mitochondrial function in influencing feeding behaviour is lesser known. Here, we used common minnows (*Phoxinus phoxinus*) to examine how liver and muscle mitochondrial function relates to the maximum amount of food consumed per meal by an individual and the time taken for appetite to return. Both the maximum meal size and appetite recovery time were positively related to muscle mitochondrial net phosphorylation efficiency. Appetite return time was also related to the maximum rate of oxidative phosphorylation; however, the relationship was positive in liver but negative in muscle. Our study shows that muscle mitochondrial efficiency influences feeding behaviours, where more efficient individuals can eat more, and eat less often. Identifying why certain individuals can consume more and return their appetite sooner may improve predictions of how individuals or populations of fish respond to food scarcity and trophic collapses.

## 1. Introduction

Variation exists naturally in the mitochondrial energetic production efficiency of individual animals and has so far been established to influence many facets of performance including growth [1,2], metabolism [3] and feed efficiency [4–6], which refers to the ratio of mass gained over the amount of food consumed [7,8]. These performance indicators are intrinsically linked to food consumption and processing, both of which are energetically costly activities that are critically important for animal survival; this is especially the case in the face of the predicted decreases in food availability and energy flow between trophic levels due to climate change [9–11].

Many relationships exist between metabolism and feeding behaviour which most likely stem from the phenomenon known as the specific dynamic action, whereby an individual’s metabolic rate increases following the consumption of food to support the processing of this meal [12,13]. These relationships include metabolic rate playing key roles in determining the lower and upper limits on meal size, the rate of digestion and subsequently the return of appetite [14–17].

The metabolic rate of an individual has been shown to be influenced by the functioning of various mitochondrial traits including mitochondrial proton leak, cytochrome *c* oxidase activity and other metabolic enzymatic activity [3,18–20]. So, considering the associations between metabolic rate and feeding behaviour, and metabolic rate and mitochondrial functioning, it can be hypothesized that the mitochondria themselves may also be playing a role in determining the limits on meal size, digestion and appetite. Few studies exist that directly investigate this hypothesis; however, there is growing evidence to support the theory that mitochondrial energy production efficiency may play a key role in driving an individual’s capacity for food processing or consumption [1,2,21].

Studies of domesticated livestock species have identified many links between feed efficiency and different traits related to mitochondrial energetic efficiency, including oxidative phosphorylation (OXPHOS) rates, electron leak and respiratory chain coupling efficiency [4,6,22,23]. However, despite these links between mitochondrial energetic efficiency and feed efficiency in domesticated animals, evidence for a similar relationship in wild species is limited.

Feeding behaviours like satiation, the state reached following a meal that prevents further consumption of food [24], and the return of appetite are often used as indicators of an individual’s capacity for food consumption and processing [25,26]. The commercial value of farmed fish and high cost of their feed has led to great interest in uncovering the factors that control and influence appetite in these species [27–29]; however, understanding the role that mitochondrial function plays in this control and influence of fish food consumption remains limited.

To date, two key studies on brown trout (*Salmo trutta*) have identified a positive relationship between an individual’s capacity for food intake and the energetic efficiency of the mitochondria, providing some of the first evidence for a relationship between mitochondrial function and food intake in a wild fish species [1,21]. In both studies, however, this relationship was found to exist only at a temperature towards the upper limit of the species and not at the lower temperature tested. What remains unclear is if this relationship exists in other fish species under energy-demanding conditions, such as after the consumption of a meal when the specific dynamic action is occurring, or if this relationship is a temperature-specific phenomenon.

Here, we examine whether variation in the capacity for both food consumption and processing is associated with variation in mitochondrial function using two tissue types that are predominantly used in similar mitochondrial function studies due to their highly metabolic nature. To achieve this, we related the maximum meal size consumed by an individual common minnow (*Phoxinus phoxinus*) and the time taken for its appetite to return after this maximum meal to the phosphorylation efficiency (*P*_efficiency_) and maximum OXPHOS respiration (*P*_PMGS_) rate of its liver and muscle mitochondria. Since the roles of these tissues vary, with the liver predominantly associated with the processing of energy and the muscle associated more with the utilization of energy, it is possible that different relationships could exist between maximum mitochondrial capacity and feeding behaviours in the two tissues as more mitochondrial activity in the muscle may require a greater quantity of food to support function. Nevertheless, we hypothesize that fish with mitochondria that are more energetically efficient (i.e. that generate a greater quantity of adenosine triphosphate (ATP) in proportion to the oxygen they have consumed) from both tissues would be more efficient at handling, processing and digesting food, and so would have a larger maximum meal size and quicker return of appetite.

## 2. Methods

### 2.1. Animal collection and husbandry

Common minnows were captured from the River Kelvin in Glasgow. To acclimatize them to laboratory conditions, they were then housed for several months in groups of 30−40 fish in dechlorinated tap water stock tanks at the University of Glasgow at 12°C, where they were fed ad libitum once per day with bloodworms (*Chironomidae*). All animal husbandry and subsequent experiments were undertaken under UK Home Office project license P89482164. At the start of the experiment, 32 fish (individual sex and age unknown) were selected semi-randomly so as to be of similar weight (2.67 ± 0.11 (s.e.m.) g). These selected fish were then housed individually in compartments of a recirculating stream tank system (each compartment being *L*: 20 cm × *W*: 12 cm, water depth 15.5 cm, water temperature 12°C (12.1 ± 0.06 (s.e.m.)°C), mean flow rate 1.7 cm s^−1^; maximum volume in the recirculation system is 300 l). This allowed for the measurement of the quantity of food consumed by each individual, but each fish was able to see its neighbours through the mesh partitions at the upstream and downstream ends of each compartment. Measurement of mitochondrial function must be conducted on fresh tissue, and there is a limit on the number of tissue samples that can be assayed per day (a maximum of six samples). Therefore, fish started the experiment sequentially rather than simultaneously, so that the time on the feeding trial and the interval between the feeding trial and the measurement of mitochondrial function was standardized. To do this, fish were moved to their individual compartments in four separate batches (group 1, *n* = 9; group 2, *n* = 9; group 3, *n* = 6; group 4, *n* = 8, total number of fish = 32) over a period of eight weeks. The number of fish in each group varied both because of this maximum limit on sampling and also based on the availability of the shared equipment in the laboratory. The groups containing more than six fish were further split into two groups, with one group starting the feeding trial first, and the second group starting the feeding trial 2 days later in order to stagger the measurement of mitochondrial function. Therefore, fish were acclimatized in their individual compartments for either 5 or 7 days prior to the start of the feeding trial. During this acclimatization period, fish were fed bloodworms (*Chironomidae*) (AQUADIP B.V. frozen bloodworm 1 kg pack) ad libitum daily, with the bloodworms being released onto the base of the compartment using a plastic pipette. This accustomed the fish to the same feeding method as would be used in the feeding trial; by the end of the acclimatization period, all fish gravitated towards the pipette to receive food when the pipette was introduced to the compartment and began eating as soon as the bloodworms were deposited into the compartment.

### 2.2. Feeding trials

Prior to feeding trials, bloodworms were defrosted in copper-free water, blotted to remove excess water, then pipetted into aliquots to be used in the feeding trials. These aliquots were weighed in numbered Eppendorf tubes (to the nearest 0.001 g) on an electronic balance (E2000D, Sartorius, Göttingen, Germany) aiming for approximately 0.1 g aliquots (0.124 ± 0.001 (s.e.m.) g) for the maximum meal size feeding trial and 0.05 g aliquots (0.053 ± 0.0003 (s.e.m.) g) for the return of appetite feeding trial and then refrozen until required for the feeding trials. These bloodworm aliquots were resuspended and defrosted in copper-free water before being used in the feeding trials.

Each fish was first given a practice feeding trial to accustom it to the methods that would be used in the real feeding trial. After this practice, trial fish were fasted for the remainder of that day and the following one and then given the real feeding trial the day after that. This ensured that there was a minimum of 24 h fasting prior to the first feeding trial.

Each feeding trial consisted of a measurement of the maximum size of meal that the fish could eat, followed by measurement of the time then taken for its appetite to return. To determine maximum meal size, fish were fed using the pre-weighed aliquots (at around 8.00), recording the ID number (and hence, known food mass) of any aliquots dispensed into the compartment. These aliquots of bloodworms were dispensed into the compartment one at a time as described above. If the entirety of this aliquot was not immediately consumed, the remaining food was left in the compartment until the other fish in this group completed the feeding trial to provide the opportunity for this food to be consumed. After this point, if there were still bloodworms remaining, these were removed from the tank, blotted to remove excess water, and weighed to allow calculation of the total mass of bloodworms consumed by each fish. If the entire aliquot was consumed, then an additional aliquot was dispensed into the compartment. This process was repeated until fish stopped feeding (i.e. did not consume any further bloodworms for at least 5 min). Meal duration time was not recorded; however, fish were fed simultaneously and so were given a similar time frame to consume the food provided for them.

The time of day that each fish stopped feeding was recorded as being the time of having eaten the maximum meal size (for future calculation of time taken until return of appetite). Fifteen minutes after this recorded time, an additional aliquot was given to confirm whether individuals had in fact reached satiation. If any of these bloodworms were consumed, then the above process to record the quantity of food consumed was repeated until feeding stopped, and the time noted.

To determine the time taken for appetite to return, food (one approx. 0.05 g bloodworm aliquot) was initially reintroduced to each fish 4 h (based on the assumption of consuming the maximum meal size and information from our pilot study and a previous study [30]) after the recorded time point at which the maximum meal size was consumed. If any of the bloodworms were consumed, then it was recorded that appetite had returned. If none of this initial aliquot was consumed within 5 min, then this food was removed from the tank and the process was repeated every hour until the first food item was consumed. The meal size consumed at this point of return of appetite was also recorded (see electronic supplementary material).

Afterwards, no further food was provided for the remainder of that day, and none was given the following day. The entire feeding trial protocol, as described above, was then repeated after the day of fasting, when we assumed that the specific dynamic action had ended so as to return energy expenditure to baseline levels; however, we did not perform any tests to officially confirm this. This was repeated once more so that in total the complete feeding trial was replicated three times for each fish.

Following the final feeding trial, fish were fasted overnight and euthanized the following day using an overdose of benzocaine (1 g l^−1^) and confirmation by destruction of the brain. Fish were weighed (to the nearest 0.001 g) on an electronic balance (E2000D, Sartorius, Göttingen, Germany), and standard length was measured (to the nearest 0.1 mm) using a calliper. Liver and muscle tissues were excised, weighed (to the nearest 0.001 g), and transferred to 2 ml of ice-cold isolation buffer (100 mM sucrose, 50 mM Tris, 5 mM MgCl_2_, 5 mM EGTA, 100 mM KCl, 1 mM ATP and pH 7.4).

### 2.3. Measurement of mitochondrial function

Tissue samples were gently homogenized by going through six passes in a dounce homogenizer at approximately 100 r.p.m. (Cole-Parmer PTFE Tissue Grinder, Cambridgeshire, UK) and were spun in a centrifuge (Micro Star 17R, VWR) at 1000*g* for 10 min at 12°C. The supernatant was transferred to a clean Eppendorf tube and spun at 8700*g* for 10 min at 12°C. The supernatant was discarded, and the pellet was resuspended in 1 ml of ice-cold storage buffer (0.5 mM EGTA, 3 mM MgCl_2_, 60 mM potassium methanesulfonate, 20 mM taurine, 10 mM KH_2_PO_4_, 20 mM Hepes, 110 mM sucrose, 0.02 mM vitamin E succinate, 2 mM pyruvate, 2 mM malate and pH 7.1).

Mitochondrial function was measured (using a modified protocol from Dawson *et al*. [1] using a high-resolution respirometer (Oxygraph-2k with O2k-Fluorescence module; Oroboros Instruments, Innsbruck, Austria)) at the acclimatization temperature of 12°C under continuous stirring. Oxygen concentration (nmol ml^−1^) within the chambers was recorded using DatLab software (Oroboros Instruments, Innsbruck, Austria). Liver (0.44 ± 0.029 (s.e.m.) mg ml^−1^) and muscle (0.34 ± 0.04 (s.e.m.) mg ml^−1^) tissue samples were transferred to separate chambers and allowed to sit for 5 min with the stirrer on to allow for a stabilization in the rate of oxygen consumption.

First malate (2 mM) and pyruvate (5 mM) were added to provide the first measure of leak state respiration (*L*_N_), followed by the addition of ADP (5 mM) to stimulate OXPHOS respiration via complex I (*P*_PM_). Glutamate (10 mM) was added next to measure maximal OXPHOS respiration rates supported via complex I in the electron transport chain (ETC) (*P*_PMG_), followed by the addition of succinate (25 mM) to obtain maximal OXPHOS respiration rates from both complexes I and II (*P*_PMGS_). Exogenous cytochrome *c* (10 mM) was added to assess the viability of the tissue samples through testing mitochondrial outer membrane integrity (severe changes in respiration rate upon addition of cytochrome *c* would indicate poor integrity [31], 28 ± 0.04 (s.e.m.)% increase in respiration rate in liver and 25 ± 0.02 (s.e.m.)% increase in respiration rate in muscle tissue). Oligomycin (10 nM) was then added to inhibit F1F0 ATP synthase and provide a second measure of leak state respiration (*L*_Omy_). Antimycin A (5 µM) addition identified the rate of non-mitochondrial oxygen consumption, with the value subsequently being subtracted from all other mitochondrial measurements. Electron donors ascorbate (2 mM) and TMPD (0.5 mM) were added last to test the maximal respiration capacity of complex IV in the ETC.

The respiratory control ratio (RCR, often used as a measure of the integrity of the mitochondrial sample preparation) was calculated as the ratio of respiration rate following the addition of pyruvate, malate, ADP, glutamate and succinate (*P*_PMGS_) over leak respiration rate following the addition of oligomycin (*L*_Omy_). The following equation was then used to calculate net phosphorylation efficiency (*P*_efficiency_):


$$P_{efficiency}=1–(1/RCR).$$

*P*_efficiency_ hypothetically ranges between zero and one (i.e. 0–100% of the mitochondrial oxygen consumption being associated with OXPHOS, higher values indicating a greater emphasis on OXPHOS rather than leak respiration). *P*_efficiency_ and maximal OXPHOS (*P*_PMGS_) respiration rate (pmol s^−1^ mg^−1^) were used as the measures of mitochondrial function in the statistical analyses (see below), but a summary of results for all the steps in the mitochondrial assay are given in electronic supplementary material, table S1.

### 2.4. Protein assay

Protein concentration was measured in a 96-well microplate using the Bradford protein assay (Bio-Rad Laboratories, Watford, UK) to standardize the amount of mitochondrial protein in each sample. The samples were assayed in a non-diluted state (excluding muscle sample from fish ID13, which was assayed at a 1 : 2 dilution, following the initial assay, to fall within the linear portion of the standard curve) along with BSA standards in the range of 0−0.8 μg μl^−1^. Standards and samples were assayed in triplicate with 10 μl of standard or sample added to each well and 190 μl of 1 : 4 dye reagent. Absorbance was measured at 595 nm. Using average absorbance from the standards, a standard curve was created to determine the protein concentration of each sample (adjusting for the dilution factor in the muscle sample from fish ID13). Protein concentration was used to normalize the values for mitochondrial function to account for the variation in the weight of each liver and muscle tissue sample from each fish.

### 2.5. Statistical analysis

The final dataset contained results from 28 individuals (results from four of the initial 32 fish were excluded due to a technical error (see electronic supplementary material ‘Exclusion of Data Points’ for more details)). Mean values from the three maximum meal size and the three time until return of appetite feeding trials were calculated for most fish and used in the statistical analyses. However, for some individuals, mean values could only be calculated from two feeding trials due to unrelated logistical problems (see electronic supplementary material ‘Exclusion of Data Points’ for more details).

A linear mixed model (R v. 4.4.1), from package lme4 (v. 1.1.35.4), was used to determine the relationship between individual variation in the measures of mitochondrial traits (*P*_efficiency_ and maximal OXPHOS rate; *P*_PMGS_) and maximum meal size in both liver and muscle. Maximum meal size was modelled as a percentage of body mass (termed ‘relative maximum meal size’) to account for any variation explained by differences in body mass. The full model for relative maximum meal size included *P*_efficiency_ and maximal OXPHOS (*P*_PMGS_) rate of both liver and muscle as covariates, and group batch number as a random effect to control for the order and group in which fish were tested. The maximal OXPHOS (*P*_PMGS_) rate covariate was log transformed within the model.

A generalized linear mixed model (R v. 4.4.1), from package glmmTMB (v. 1.1.9), was used to determine the relationships between individual variation in measures of mitochondrial traits (*P*_efficiency_ and maximal OXPHOS (*P*_PMGS_) rates) and the time taken until the return of appetite in both liver and muscle. This model assumed a gamma distribution as the data was positively skewed. The full model for time until return of appetite included *P*_efficiency_ and maximal OXPHOS (*P*_PMGS_) rate of both liver and muscle and relative maximum meal size as covariates, and interactions between relative maximum meal size and both *P*_efficiency_ and maximal OXPHOS (*P*_PMGS_) rate as covariates. The maximal OXPHOS (*P*_PMGS_) rate covariate was log transformed within the model. Fish mass was not included in this model as this variable was nested into the relative maximum meal size covariate. Group batch number was again included as a random effect. Models were simplified using likelihood ratio tests, from package lmtest (v. 0.9.40), by sequentially removing terms, starting with interactions, and then retesting for model significance after the removal of each term (see electronic supplementary material for final models).

The correlation between the measures of mitochondrial function (either *P*_efficiency_ or *P*_PMGS_) in the liver and muscle tissues was evaluated using Spearman’s rank correlation. These correlation tests were also performed for the other mitochondrial function traits measured. The repeatability of both the measures of maximum meal size and time taken until the return of appetite for the fish sampled in this study was calculated using the lmm method for repeatability estimation from package rptR (v. 0.9.22) (see electronic supplementary material, table S2).

## 3. Results

### 3.1. Effects of mitochondrial energetic efficiency on maximum meal size

**Figure 1.**
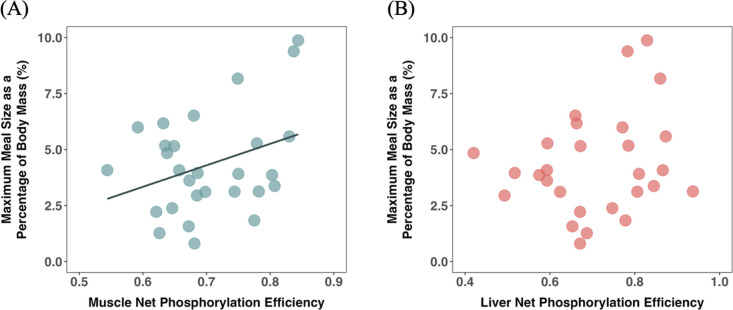
Relationship between maximum meal size as a percentage of body mass (%) and net phosphorylation efficiency (*P*_efficiency_) of mitochondria in (A) the muscle (correlation coefficient = 0.34) and (B) the liver in common minnows (*n* = 28). The solid line shows the significant effect of net phosphorylation efficiency of the muscle mitochondria; see table 1 for statistical analyses.

While body mass was not found to be a significant predictor of maximum meal size (slope = 0.024, *t* = 1.213, d.f. = 26, *p =* 0.24), maximum meal size was nonetheless modelled as a percentage of body mass (termed ‘relative maximum meal size’) to account for any variation explained by differences in body mass. The net phosphorylation efficiency (*P*_efficiency_) of the mitochondria in the muscle was found to be a significant predictor of relative maximum meal size (*F* = 6.93, d.f. = 1, 20.17, *p =* 0.016), with higher *P*_efficiencies_ being associated with greater relative maximum meal sizes (figure 1A and table 1). However, among-individual variation in relative maximum meal size was not predicted by the *P*_efficiency_ of the liver mitochondria (*p =* 0.74; figure 1B and table 1), nor the maximum OXPHOS (*P*_PMGS_) rates of either the liver or muscle mitochondria (*p >* 0.1; table 1).

**Table 1.** Results from generalized linear mixed model analyses of the factors influencing relative maximum meal size (i.e. the maximum mass of food consumed in one meal, relative to body mass) in common minnows (*n* = 28). Note: group batch number was included as a random effect to control for the order and group in which fish were tested. Bold denotes significant terms.

dependent variable	parameter	parameter estimate ± s.e.	*F*	d.f.	*p*‐value
relative maximum meal size	liver *P*_efficiency_	−0.91 ± 2.83	0.11	1, 22.72	0.74
	log(liver OXPHOS (*P*_PMGS_))	0.91 ± 0.62	2.28	1, 22.57	0.14
	**muscle *P*_efficiency_**	**11.64** ± **4.5**	**6.93**	**1, 20.17**	**0.016**
	log(muscle OXPHOS (*P*_PMGS_))	−0.48 ± 0.7	0.6	1, 21	0.45

### 3.2. Effects of mitochondrial energetic efficiency on time until return of appetite

**Figure 2.**
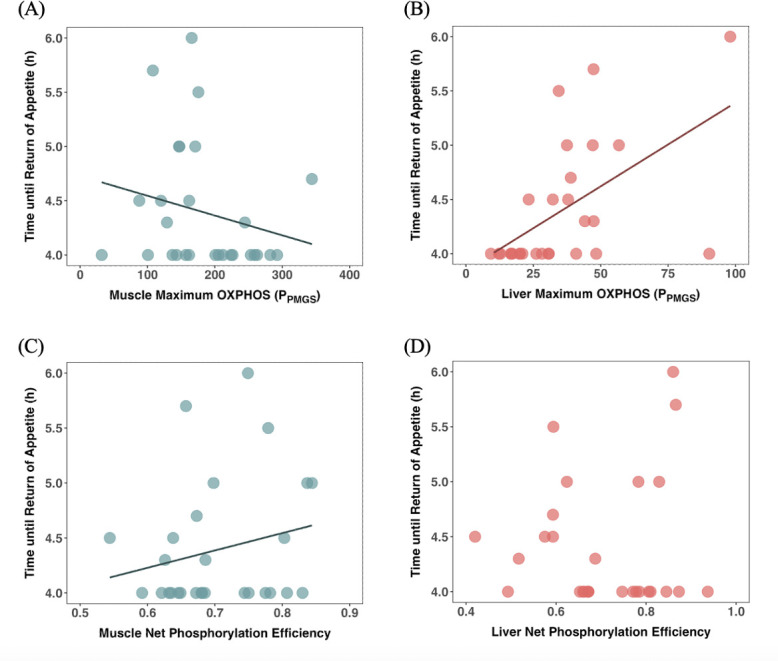
Relationship between the time taken until the return of appetite (hours) and maximal OXPHOS (*P*_PMGS_) respiration rate (pmol s^−1^ mg^−1^) in (A) the muscle mitochondria (correlation coefficient = −0.22) and (B) the liver mitochondria (correlation coefficient = 0.55), and net phosphorylation efficiency (*P*_efficiency_) of (C) the muscle mitochondria (correlation coefficient = 0.22) and (D) the liver mitochondria in common minnows (*n* = 28). The solid lines show significant effects; see table 2 for statistical analyses.

Relative maximum meal size influenced the time until return of appetite (*z* = −2.13, *p =* 0.03), as fish that ate larger meals for their body size resumed feeding earlier (table 2). This indicates physiological differences among fish in their capacity to process food, and indeed the time until appetite had returned was also predicted by maximal OXPHOS (*P*_PMGS_) rate of both the muscle (*z* = −2.45, *p =* 0.01) and liver (*z* = 4.17, *p <* 0.0001). However, this was in opposite directions, as a shorter time to return of appetite was associated with a higher muscle *P*_PMGS_ rate (−0.31 ± 0.13) but a lower liver *P*_PMGS_ rate (0.15 ± 0.04) (figure 2A,B, and table 2). Variation in the time until the return of appetite was also predicted by net phosphorylation efficiency (*P*_efficiency_) of the muscle mitochondria (*z* = 2.86, *p =* 0.004), but in the opposite direction to that predicted: higher efficiency was associated with greater times until the return of appetite (figure 2C and table 2). There was also a significant interaction between relative maximum meal size and muscle maximum OXPHOS (*P*_PMGS_) rate, so that the effect of maximum meal size on time until return of appetite was affected by muscle OXPHOS (*P*_PMGS_) rate. For analysis of the meal size consumed at this point where appetite returned, see electronic supplementary material, table S3.

**Table 2.** Results from generalized linear mixed model analyses of the factors influencing the time until return of appetite in common minnows (*n* = 28). Note: group batch number was included as a random effect to control for the order and group in which fish were tested. Bold denotes significant terms. Initial model: time until return of appetite = liver *P*_efficiency_ + muscle *P*_efficiency_ + log(liver *P*_PMGS_) + log(muscle *P*_PMGS_) + relative maximum meal size + relative maximum meal size × log(muscle *P*_PMGS_) + relative maximum meal size × liver *P*_efficiency_ + relative maximum meal size × muscle *P*_efficiency_ + relative maximum meal size × log(liver *P*_PMGS_)

dependent variable	parameter	parameter estimate ± s.e.	*z-*value	*p*‐value
time until return of appetite	liver *P*_efficiency_	−0.1 ± 0.13	−0.75	0.46
	**log(liver OXPHOS (*P*_PMGS_))**	**0.15** ± **0.04**	**4.17**	***p* < 0.0001**
	**muscle *P*_efficiency_**	**0.81** ± **0.28**	**2.86**	**0.004**
	**log(muscle OXPHOS (P_PMGS_))**	**−0.31** ± **0.13**	**−2.45**	**0.01**
	**relative maximum meal size**	**−0.27** ± **0.12**	**−2.13**	**0.03**
	**relative maximum meal size × log(muscle OXPHOS (*P*_PMGS_))**	**0.05** ± **0.02**	**2.12**	**0.03**

### 3.3. Mitochondrial energetic efficiency traits

There was no correlation between the liver and muscle measurements for any of the mitochondrial function traits from the same fish (see table 3 for analysis of *P*_efficiency_ and *P*_PMGS_ and electronic supplementary material, table S1 for that of other mitochondrial states). However, the correlation between muscle and liver *P*_efficiency_ showed a positive trend (*p =* 0.060). The rates of both *P*_PMGS_ and *L*_Omy_ were found to be significant predictors of the net phosphorylation efficiency (*P*_efficiency_), calculated using these *P*_PMGS_ and *L*_Omy_ rates, of the mitochondria in both the liver and muscle (see electronic supplementary material, table S4).

**Table 3.** Key mitochondrial properties (maximum OXPHOS (*P*_PMGS_) respiration rate and net phosphorylation efficiency (*P*_efficiency_)) of muscle and liver from common minnows (*n* = 28), including the correlation between liver and muscle measurements from the same individual.

parameter	tissue	mean ± s.e.m.	spearman’s *ρ*	*p*‐value
*P*_PMGS_	liver	35.2 ± 3.97 (pmol s^−1^ mg^-1^)	−0.24	0.21
	muscle	183.9 ± 13.28 (pmol s^−1^ mg^-1^)		
*P*_efficiency_	liver	0.71 ± 0.02	0.36	0.06
	muscle	0.7 ± 0.02		

## 4. Discussion

The results from our study suggest that feeding capacity and processing behaviours can be predicted from the efficiency of mitochondrial traits in both the liver and muscle, but that these relationships vary between tissues. Muscle net phosphorylation efficiency was found to be positively related to both the maximum capacity and rate of processing of food, such that fish whose muscle mitochondria had high phosphorylation efficiencies were able to consume more food in one meal and took longer to resume feeding. However, while muscle maximum OXPHOS (*P*_PMGS_) rate was found to be negatively related to the time taken for appetite to return, this was the opposite relation to that in the liver, where the maximum OXPHOS (*P*_PMGS_) rate was found to be positively related to the time taken until the return of appetite.

### 4.1. Maximum food intake is predicted by muscle net phosphorylation efficiency

Our study found that the among-individual variation in net phosphorylation efficiency (i.e. the relative proportion of mitochondrial respiration that was devoted to OXPHOS rather than offsetting the proton leak) of the mitochondria found in the myotome muscles explained a significant amount of the variation in maximum food intake: specifically fish that had a greater net phosphorylation efficiency in their muscle mitochondria consumed a greater maximum meal size in proportion to their body mass. To date, there are few previous studies that have explicitly examined a relationship between mitochondrial function and food intake, despite the two being inherently linked. Instead, most studies focus on the link between mitochondrial traits and growth performance [1,2,32,33]. However, there are two studies on juvenile brown trout that have reported associations between mitochondrial function and food intake, whereby the individuals who had the highest daily food intake were those that had higher mitochondrial respiratory capacities in the liver [1], and lower mitochondrial leak respiration rates in both the liver and muscle [21]. Both of these studies also found these relationships to exist only at a higher temperature (19°C in [21] and 19.5°C in [1]). Our study did not focus on investigating responses to elevated temperatures but instead tested the relationships between mitochondrial function and feeding in common minnows held at a temperature within their natural range (12°C). Despite this, we identified related effects of mitochondrial function on food intake, albeit only in muscle mitochondria.

Our findings concur with previous findings to suggest that the energetic production efficiency of the mitochondria play at least a partial role in modulating an individual’s capacity for food consumption [1,21]. More importantly, we found this relationship to exist only in the muscle tissue. This may simply be a result of individuals who have more energetically efficient muscle mitochondria in turn requiring a greater intake of food to power the efficient production of energy within this tissue. However, it is also important to consider the optimal environment in which mitochondrial function was measured, with a surplus of substrates available. Therefore, individuals with more efficient mitochondria may devote less resources to sustain muscle activity, allowing fish to devote more energy to processing food, and ultimately enabling them to eat more.

Despite this, the identification of similar relationships in the two previous studies at a warmer temperature, and in both the liver and muscle tissues, suggests that perhaps these associations between mitochondrial energetic efficiency and feeding behaviour that exist across different tissues may vary with temperature, possibly favouring muscle energy usage in colder temperatures and liver processing in warmer temperatures. More specifically, for temperate freshwater species like the common minnow, it may be that during periods of colder temperatures and thus decreased food availability, energy production is allocated predominantly to muscle tissue to support whole-body activity, and production is reduced in the liver due to a decrease in food consumption. However, in periods of warmer temperatures and thus increased food availability, energy production is distributed more evenly, and the liver plays a more predominant role to support the processing and storage of energy substrates from the increased consumption of food. Therefore, individuals held in colder temperatures may exhibit greater associations between mitochondrial energetic efficiency and feeding behaviour in the muscle tissue and instead exhibit associations in both the muscle and liver tissues when held in warmer temperatures.

The specific dynamic action of an individual, that is, the increase in energy expenditure and metabolic rate that is required to fuel the processes associated with the digestion of a meal [12,13], should also be considered when studying an individual’s capacity for food consumption. The specific dynamic action increases with the size of a meal, so the ability of an individual to increase its meal size will also depend on the energetic efficiency of that individual to digest a meal. This has previously been identified as a trade-off that fish need to face in regard to meal size, between the growth benefits of consuming a large meal and the energy sparing benefits of consuming a smaller meal that does not require such a large specific dynamic action [34].

### 4.2. Time until return of appetite is predicted by liver and muscle mitochondrial traits

Liver and muscle maximum OXPHOS (*P*_PMGS_) rates were both significant, but opposite, predictors of the time taken until the return of appetite, along with muscle net phosphorylation efficiency. In fish, the liver plays important roles in processes related to digestion including the metabolism of carbohydrates, proteins and lipids [35], as well as the synthesis of enzymes required for digestion and the production and excretion of bile [36]. It can therefore be assumed that a greater food processing capacity in the liver may in turn require a greater mitochondrial capacity for energy production to fuel this capacity. However, it is likely that fish vary not only in the amount of food eaten, but also the extent to which it is digested (i.e. the assimilation efficiency): fish may be slower to resume feeding if they extract more of the nutrients out of the previous meal, due to a trade-off between gut passage time and assimilation efficiency [37]. This greater assimilation efficiency would come at a short-term cost since it would require a greater energetic expenditure during the period of digesting a meal, but it would then generate more useable resources per unit of food ingested [38]. This may explain the positive relationship between liver OXPHOS (*P*_PMGS_) capacity and time to return of appetite, if in fact fish with a high liver OXPHOS capacity were using this to extract more from their food.

While neither gut passage time nor assimilation efficiency were measured in the present study, there is some evidence from other contexts that intraspecific variation in digestive traits may be linked to mitochondrial function. The concept of feed efficiency (the proportion of ingested food that is converted to growth, and thus an indirect proxy for assimilation efficiency) has been much studied in domesticated livestock, due to its clear importance in determining farming efficiency. In chickens, the muscle mitochondria from high feed efficiency individuals have been found to exhibit increased mitochondrial coupling efficiency, greater maximum OXPHOS rates and lower electron leak than those from low feed efficiency chickens [22,23].

In our study, individuals with higher muscle *P*_efficiency_ exhibited an increased time until the return of appetite. While this result ran counter to our initial prediction, it may be because these individuals are more efficient at utilizing the food they have consumed.

Given that the muscles are the largest tissue in the body (and so have a major impact on the overall energy budget), this would mean that fish with efficient muscle mitochondria would be slower to deplete the circulating pool of nutrients, leading to a later triggering of the nutrient-sensing mechanisms that prompt a resumption of appetite: it should be noted that the timing of the return of appetite will be influenced by the rate of energy depletion as well as the speed of digesting the last meal [39,40].

Finally, it should be noted that the analysis also revealed a significant interaction, such that the effect of muscle maximum OXPHOS (*P*_PMGS_) rate on the time to return of appetite depended on the maximum amount of food consumed in the previous meal. It is likely that the specific dynamic action may be playing a role in this interaction, since the speed and ability of an individual to digest a given maximum meal size will depend on its ability to raise its metabolic rate to support this specific dynamic action. However, this interaction is difficult to interpret, and so further experiments would be required to validate its existence and tease apart its meaning.

### 4.3. The association between feeding behaviours and mitochondrial function

While we identified multiple associations between mitochondrial energetic efficiency and specific feeding behaviours in common minnows, it is important to consider that other factors will also be at play in determining these relationships that could not be accounted for during this study. Mitochondrial energetic efficiency is connected to an individual’s metabolic rate. In turn, metabolic rate and the specific dynamic action itself are inherently linked to an individual’s feeding behaviour, including its feeding capacity and meal processing times [14,16,41,42]. Furthermore, various relationships have been identified between metabolic rate and mitochondrial traits [3,20,43], so it is likely that the energetic efficiency of the mitochondria is exerting a role in determining an individual’s capacity for different feeding behaviours directly. So, while our study supports this idea and has identified associations between mitochondrial energetic efficiency and maximum food intake and return of appetite feeding behaviours, many other relationships and external factors are still at play that may be influencing the nature of these associations, such as food availability, energy expenditure, stressors and social hierarchies [44–46]. It should also be considered that this relationship may also vary with diet; our study used bloodworms as the sole food source for the fish; however, factors like the nutrient and calorie content of different food sources may influence the associations between feeding behaviour and mitochondrial energetic efficiency identified in our study.

Nonetheless, understanding the factors that influence how much food an individual is capable of consuming, how efficient they are at processing this meal, and how soon they need to feed again is crucial to help predict how different individuals will respond to shifts in the availability of food. Our study aids in this quest to determine the outcomes of feeding behaviour by uncovering that mitochondria do in fact play a role in influencing food intake even in non-challenging conditions (e.g. ad libitum food supply and benign temperatures), although these relationships differ between tissues. Identifying the existence of these relationships, and defining the nature of them, will become crucial in aiding our understanding of the mechanisms that influence feeding behaviour. More significantly, it is critical to understand how ectothermic species may be capable of responding to, and surviving, periods of decreased food availability, especially in the face of predicted collapses of trophic food webs and diminished food availability as a consequence of climate change.

## Data Availability

All data is included in electronic supplementary file #2 [47].
